# Efficacy of Quanduzhong capsules in regulating blood pressure circadian rhythm in essential hypertension: a multicenter, randomized, add-on controlled clinical trial

**DOI:** 10.3389/fphar.2025.1659073

**Published:** 2025-10-08

**Authors:** Bi-Hua Tang, Cong-Hui Zhou, Run-Ming He, Zi-Yun Li, Jin-Cheng Wang, Yu-Ting Cui, Jia-Xi Zou, Jun-Ping Xiao, Zhi-Gang Zhou, Xiao-Feng Li, Yan Yang, Jing-Qing Hu

**Affiliations:** ^1^ Affiliated Hospital of Integrated Traditional Chinese and Western Medicine, Nanjing University of Chinese Medicine, Nanjing, China; ^2^ School of Acupuncture and Tuina, School of Regimen and Rehabilitation, Nanjing University of Chinese Medicine, Nanjing, China; ^3^ Institute of Basic Theory for Chinese Medicine, China Academy of Chinese Medicine Science, Beijing, China; ^4^ Jiangsu Province Hospital of TCM, Affiliated Hospital of Nanjing University of Chinese Medicine, Nanjing, China; ^5^ Hospital of Chengdu University of Traditional Chinese Medicine, Chengdu, China; ^6^ Jiangxi Puzheng Pharmaceutical Co, Ltd., Jian, China; ^7^ China Science and Technology Development Center for Chinese Medicine, Beijing, China; ^8^ Tianjin University of Traditional Chinese Medicine, Tianjin, China

**Keywords:** Quanduzhong capsules, essential hypertension, blood pressure variability, circadian rhythm, clinical trial

## Abstract

**Background:**

Abnormal circadian rhythm of blood pressure is recognized as an independent risk factor for target organ damage in the heart, brain, and kidneys. *Eucommia ulmoides* Oliv *[Eucommiaceae, Eucommiae cortex]*, a traditional Chinese medicine, has been reported to exhibit antihypertensive effects and may regulate blood pressure variability. This study aimed to evaluate the efficacy and safety of Quanduzhong capsules in regulating the circadian rhythm of blood pressure in patients with essential hypertension.

**Methods:**

We designed a randomized controlled clinical trial. A total of 136 participants who had essential hypertension and abnormal circadian rhythm of blood pressure were randomly assigned to the test or control group, each comprising 68 individuals, using the random number table method. Both groups maintained their original Western antihypertensive medicine regimen, with the test group receiving additional treatment of Quanduzhong capsules (administered twice daily, 1.48 g each time). The treatment duration for both groups was 12 weeks. The patients were visited at baseline and at the end of the 4th, 8th, and 12th weeks of the intervention. Their 24-h ambulatory blood pressure and clinic blood pressure were measured at baseline and the end of 12th weeks. Primary outcomes included the recovery rate of dipper blood pressure rhythm, the standard deviation of blood pressure (SD), and the coefficient of variation of blood pressure (CV).

**Results:**

After 12 weeks of treatment, the results in the full analysis set and in the per-protocol set showed no statistically significant differences between the treatment group and the control group in SD and CV. However, among the subjects who did not use calcium channel blockers, the treatment group demonstrated significantly better improvements in 24-h standard deviation of diastolic blood pressure (*P < 0.05*) than the control group.

**Conclusion:**

Our findings suggest that Quanduzhong capsules can effectively improve the circadian rhythm of blood pressure without calcium channel blockers.

**Clinical Trial Registration:**

https://www.chictr.org.cn/showproj.html?proj=127240, identifier ChiCTR2100046830.

## 1 Background

Hypertension stands as one of the most prevalent chronic non-communicable diseases globally. Epidemiological studies reveal that hypertension is linked to approximately 10.8 million deaths annually (NCD Risk Factor Collaboration, 2021). Approximately 435 million people in China have high-normal blood pressure (National Center for Cardiovascular Diseases and The Writing Committee of the Report on Cardiovascular Health and Diseases in China, 2025), with over half exhibiting abnormalities in their circadian blood pressure rhythm (Ding et al., 2021). The circadian rhythm of normal blood pressure shows a fluctuation curve of “double peaks and one valley”. The nocturnal blood pressure is the lowest, rising to the first peak in the morning, then slightly decreasing, reaching a second peak at 4:00–6:00 p.m., then gradually decreasing again until reaching the lowest point of the day at 2:00–4:00 a.m. (Costello and Gumz, 2021; Schutte et al., 2022; Faraci and Scheer, 2024). Although the absolute blood pressure value remains crucial in cardiovascular events related to hypertension, observational studies and clinical trials suggest that these outcomes may also be influenced by increased blood pressure variability (BPV). “Non-dipper” nocturnal hypertension and morning blood pressure surges are significant predictors of adverse cardiovascular outcomes. Disruptions in the circadian rhythm of blood pressure have been identified as an independent risk factor for target organ damage in the heart, brain, and kidney (Stevens et al., 2016; Hawkes et al., 2022; Sheikh et al., 2023). Currently, Western medicine lacks a specific method to regulate abnormal blood pressure rhythms, typically relying on long-acting antihypertensive drugs or adjusting medication times (Kario et al., 2022). Therefore, it is imperative to explore novel therapeutic strategies to stabilize the circadian rhythm of blood pressure.

*Eucommia ulmoides* Oliv *[Eucommiaceae, Eucommiae cortex]*(Duzhong) is a traditional Chinese medicine, dating back thousands of years. According to the Pharmacopoeia of the People’s Republic of China 2020, Duzhong is effective at tonifying the liver and kidney and strengthening muscles, bones, and placenta. It is used to treat liver and kidney insufficiency, waist and knee pain, muscle weakness, dizziness, pregnancy bleeding, and restless fetal movement. Contemporary research (Zhang et al., 2023; Li et al., 2024) has suggested that various parts of Duzhong (bark, leaves, male flowers, and seeds) contain a diverse array of chemical metabolites, including lignans, iridoids, flavonoids, phenylpropanoids, steroids, and terpenes. *E. ulmoides* extracts/lignans lower blood pressure via multiple pathways: suppressing cAMP activity and calcium ion internalization, modulating NO and the renin–angiotensin system, and inducing vascular relaxation, while also increasing coronary flow (He et al., 2014). Consequently, Duzhong is widely utilized in various fields, including medicine, health food, feed additives, and daily chemical products. Clinical studies (Yang et al., 2021; Wang et al., 2025) have also validated the antihypertensive properties of Duzhong. Quanduzhong capsules, which contain Duzhong extract, have been shown to be effective in treating hypertension. In our previous research, we conducted a clinical investigation that used Quanduzhong capsules to treat hypertension of grade 1 (Jiang et al., 2022). Compared with the control group during the same period, the test group demonstrated significantly greater reductions in clinic systolic and diastolic blood pressure after 12 weeks of treatment, along with a higher blood pressure control rate (55.17% vs. 24.14%; χ^2^ = 5.735, *P* < 0.05), re-affirming the antihypertensive effect of Duzhong. Furthermore, the study found that after 12 weeks of treatment, 5 of 19 non-dipper hypertension patients in the test group converted to dipper patterns, compared to only 1 of 19 in the control group. Although the current analysis showed no statistically significant difference between groups due to limited sample size, these results suggest a potential trend of Quan-Duzhong capsules in regulating blood pressure circadian rhythms.

Drawing upon prior research, we identified the key metabolites in Quanduzhong capsules and conducted a randomized controlled trial to comprehensively assess the effect of Quanduzhong capsules on the circadian rhythm of blood pressure among patients with essential hypertension. A total of 136 participants were randomly assigned to the test group or the control group, with 68 individuals in each group, using the random number table method. Both groups adhered to their original Western antihypertensive medication regimen, with the test group receiving additional Quanduzhong capsules (administered twice daily, three capsules each time). The treatment period for both groups lasted 12 weeks. Monitoring procedures encompassed 24-hour ambulatory blood pressure measurements at baseline and post-treatment, as well as clinic blood pressure assessments at baseline and at 4 weeks, 8 weeks, and 12 weeks of treatment. Home blood pressure monitoring was also conducted throughout the trial. The primary outcomes focused on the recovery rate of the circadian rhythm and the standard deviation (SD), coefficient of variation (CV), mean blood pressure values, and safety indicators for both groups. The study was granted ethical approval by the Ethics Committee of the Institute of Basic Theory of Traditional Chinese Medicine, China Academy of Chinese Medical Sciences (Ethical Approval No. 2020-KY-EC-011).

## 2 Methods

### 2.1 Trial design

This multicenter, randomized, case–control trial was conducted between August and October 2021 in two prominent hospitals in mainland China: the Seventh Affiliated Hospital of Southern Medical University and Anyang Traditional Chinese Medicine Hospital. A total of 136 patients participated in this trial. The study is registered with the Chinese Clinical Trial Registry (ChiCTR2100046830) and adheres to the principles of the Declaration of Helsinki and Good Clinical Practice guidelines.

### 2.2 Diagnostic criteria

Diagnostic criteria for hypertension: Following the 2018 Chinese guidelines for the management of hypertension (Writing group of 2018 Chinese guidelines for the management of hypertension, 2019), hypertension is characterized as SBP >140 mmHg and/or DBP >90 mmHg and measured on three separate clinic visits without the influence of antihypertensive medication. Patients with a documented history of hypertension currently undergoing antihypertensive therapy should be diagnosed as hypertensive, even if their blood pressure measurements fall below 140/90 mmHg. Hypertension severity is further categorized into Grades 1, 2, and 3, depending on the degree of blood pressure elevation (Table 1).

**TABLE 1:** Classification and definition of blood pressure levels (mmHg).

Classification	Systolic pressure		Diastolic pressure
Normal blood pressure	<120	and	<80
High-normal blood pressure	120–139	and (or)	80–89
Hypertension	>140	and (or)	>90
Grade 1 (mild)	140–159	and (or)	90–99
Grade 2 (moderate)	160–179	and (or)	100–109
Grade 3 (severe)	>180	and (or)	>110
Isolated systolic hypertension	>140	and	<90

The higher grade of hypertension should be taken as the basis when systolic and diastolic blood pressure belong to different grades.

Diagnostic criteria for non-dipper blood pressure rhythms: Non-dipper blood pressure, in a narrow sense, refers to a decrease in nighttime systolic blood pressure of 0%–10%. Broadly, non-dipper blood pressure refers to all abnormal blood pressure rhythms. For the purpose of this study, we apply the broad non-dipper blood pressure rhythm criteria. The specific rhythms are defined according to the 2020 Chinese Hypertension League Guidelines on Ambulatory Blood Pressure Monitoring (Writing Group of the 2020 Chinese Hypertension League Guidelines on Ambulatory Blood Pressure Monitoring, 2021), and classified based on the percentage of nocturnal systolic blood pressure decrease (Table 2).

**TABLE 2:** Classification of blood pressure circadian rhythm.

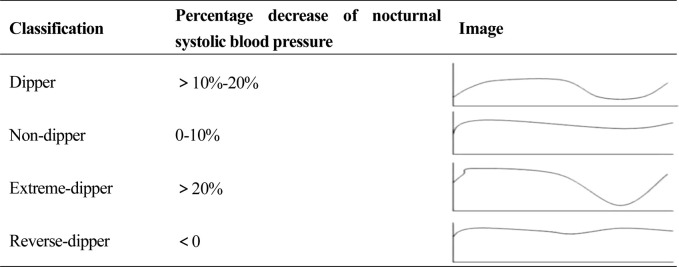

Percentage decrease of nocturnal systolic blood pressure = (mean daytime SBP - mean nighttime SBP)/mean daytime SBP × 100%. Mean daytime SBP is defined as the average SBP between 06:00 and 22:00. Mean nighttime SBP refers to the average SBP between 22:00 and 06:00.

### 2.3 Participants

Inclusion criteria: (1) Male or female patients aged between 40 and 75 years of age. (2) Essential hypertension patients. (3) Non-dipper 24-hour blood pressure rhythm patients. (4) Patients maintaining a stable (longer than 1 month) regimen of Western antihypertensive medicine. (5) Patients without other complicated diseases. (6) Patients who volunteered for the trial and provided signed informed consent.

Exclusion criteria: (1) Patients known or suspected to have hypersensitivity to the test drug or any of its metabolites. (2) Patients suffering from malignant hypertension, hypertensive emergencies, hypertensive crises, or hypertensive encephalopathy. (3) Patients presenting evidence of secondary hypertension, including but not limited to conditions such as bilateral or unilateral renal artery stenosis, polycystic kidney, aldosteronism, aortic constriction, Cushing’s syndrome, or pheochromocytoma. (4) Patients with gastrointestinal lesions or gastrointestinal surgeries potentially impacting drug absorption or excretion, such as gastrointestinal resection, active gastrointestinal inflammation, ulcers, or gastrointestinal bleeding within the past 3 months. (5) Patients leading a circadian lifestyle or demonstrating irregular sleep patterns. (6) Patients with a history of alcohol or drug abuse. (7) Patients with coexisting neurological or psychiatric disorders impeding cooperation. (8) Pregnant or lactating women or those planning for childbirth in the near future. (9) Patients with severe hepatic or renal impairment (10) Patients deemed unsuitable for trial participation at the investigator’s discretion. (11) Patients who had partaken in other clinical trials within 3 months prior to the commencement of the current trial.

Withdrawal criteria: Investigator-initiated discontinuation or withdrawal criteria: Patients who experienced serious or significant adverse events, demonstrated poor compliance, or were mistakenly included and did not meet the inclusion criteria or satisfy any of the exclusion criteria were deemed unsuitable to continue the experiment.

Participant withdrawal criteria: Patients who were unwilling or unable to proceed with the clinical trial and expressed their wish to withdraw to the investigator, discontinued medication intake and testing, or became lost to follow-up, even though they did not explicitly request to withdraw from the trial, were also considered withdrawn.

### 2.4 Randomization and intervention

A random number table method was utilized to distribute the 136 patients into the experimental and control groups at a 1:1 ratio. The random number sequence was managed by a dedicated individual within the research group, who determined the group assignment once qualified study subjects had been identified. The control group continued with their original Western antihypertensive medicine regimen, while the test group added Quanduzhong capsules, three capsules (1.48 g) twice daily, taken between 8:00–10:00 and 20:00–22:00, respectively, for a duration of 12 weeks. Quanduzhong capsules were provided free of charge by the research project team.

### 2.5 Preparation of the drug

Quanduzhong capsules contain *E. ulmoides* extract and are approved by China’s Food and Drug Administration. Each 0.48 g capsule represents 2.5 g of the crude herb. Raw materials originated from Duzhong good agricultural practices (DZ GAP) bases established by Jiangxi Puzheng Pharmaceutical Co., Ltd. in Jiangxi’s Jinggangshan and Jishui regions.

For this clinical trial, capsules were manufactured by Jiangxi Puzheng Pharmaceutical Co., Ltd (Batch: batch number: 210402; Inspection: CP054210501). Processing commenced with cork removal from 2,500 g DZ. Following pulverization, 250 g of powder was reserved. The remaining material underwent milling before 85% ethanol reflux extraction (2 h). Post-filtration, ethanol recovery occurred. The resultant liquid medicine was retained. Subsequent aqueous decoction involved dual water extractions (1 h each). Combined decoctions were filtered. Filtrate integration with retained liquid preceded vacuum concentration. This yielded a paste (1.30 g/mL relative density at 80 °C). Incorporation of the reserved 250 g powder with soluble starch followed. The final steps involved drying, grinding, sieving, and encapsulation into 1,000 units. Thus, sequential extraction employed ethanol followed by water. The crude drug powder within capsules underwent innovative micropowder processing. This ensured extract homogeneity/stability while optimizing active metabolite dispersion upon administration, maximizing the therapeutic effect. Unfilled gelatin capsules came from Suzhou Capsule Co., Ltd (Batch: 12875893).

### 2.6 Fingerprint spectrum and LC-MS/MS analysis of Quanduzhong capsules

#### 2.6.1 Fingerprint spectrum

Preparation of the reference solution: the appropriate amounts of the following reference substances, provided by the Chinese Institute for the Control of Pharmaceutical and Biological Products, were accurately weighed: paeoniflorin (Batch No. T06-20131,022), chlorogenic acid (Batch No. L11-20130502), loganin (Batch No. J04-20130113), and pinoresinol diglucoside (Batch No. S21-20130811). Solutions were prepared with concentrations of 0.321 mg/mL for paeoniflorin, 0.052 mg/mL for chlorogenic acid, 0.072 mg/mL for loganin, and 0.084 mg/mL for pinoresinol diglucoside.

Preparation of the sample solution: The Quanduzhong capsules provided by Jiangxi Puzheng Pharmaceutical Co., Ltd., consisted of a total of 16 batches. A 0.3 g sample of the product was accurately weighed and placed in a conical flask. Subsequently, 25 mL of methanol was accurately added, reweighed, and sonicated for 30 min. Any weight loss was compensated for with additional methanol, the mixture was filtered, and the filtrate was collected for further analysis.

Analysis: An Agilent LC-1260 high-performance liquid chromatograph (HPLC) was utilized for analysis and the establishment of the fingerprint spectrum. The chromatographic conditions were as follows: Column: Cosmosil column (5 μm, 4.6 × 250 mm); Wavelength: 208 nm; Flow rate: 0.8 mL/min; Column temperature: Ambient; Mobile phase: Gradient elution comprising acetonitrile and 0.1% phosphoric acid water (0–40 min, 6%–16% acetonitrile; 40–75 min, 16%–30% acetonitrile; 75–77 min, 30%–6% acetonitrile; 77–90 min, 6% acetonitrile). The reference solution and the sample solution were injected under these chromatographic conditions, and the chromatograms were recorded. Utilizing the Fingerprint Similarity Evaluation System 2004A (National Pharmacopoeia Commission), the peaks indicated on the reference chromatogram were matched, multi-point correction was performed, and the similarity was calculated.

#### 2.6.2 LC-MS/MS analysis

LC-MS/MS sample preparation: 0.3 g of Quanduzhong capsule powder was combined with 25 mL of deionized water. This mixture underwent 30 min of ultrasonic dissolution. A 1-mL aliquot was then diluted 100-fold with deionized water. Subsequent processing involved high-speed centrifugation followed by filtration through a 0.22-μm membrane (Xu et al., 2023).

Metabolite identification was performed using an ultra-performance liquid chromatography-quadrupole time-of-flight mass spectrometry (UPLC-Q-TOF MS) system (AB SCIEX TripleTOF 5600+, Foster City, CA, United States). Chromatographic separation was achieved on a C18 column (100 mm × 2.1 mm, 1.8 μm) with a flow rate of 0.25 mL/min. The mobile phase consisted of (A) acetonitrile and (B) 0.1% (v/v) formic acid in water. The gradient elution program was carried out as follows: 0–2 min, 10% A; 2–5 min, 10%–25% A; 5–15 min, 25%–40% A; 15–23 min, 40%–90% A; 23–27 min, 90% A.

An electrospray ionization (ESI) source operated in the negative ion mode. Analytes were scanned across m/z 120–1,500. Key parameters: atomization temperature: 600 °C; spray voltage: −4500 V; DP: −80 V; data acquisition used TOF-MS-IDA-MS/MS mode.

### 2.7 Outcome measures

Primary efficacy indicators: Ambulatory blood pressure monitoring (ABPM). The ABPM devices employed in this study are the Beneware ABP-021 and Hingmed WBP-02A, both of which are recommended by professional societies on their website (www.stridebp.org). The primary efficacy indicators derived from these devices are as follows: recovery rate of dipper blood pressure rhythm is defined as the proportion of subjects who restored a dipper blood pressure circadian rhythm after treatment within the cohort. A dipper blood pressure rhythm is determined by the percentage decrease in nocturnal systolic blood pressure, as outlined in Table 2. Blood pressure variability index: To assess blood pressure variability, we measured the standard deviation (SD) and coefficient of variation (CV) of blood pressure fluctuations over 24 h. These metrics were calculated for overall, diurnal, and nocturnal blood pressure separately. Other efficacy indicators: Mean 24-hour blood pressure: Daytime blood pressure (dBP) is defined as the average blood pressure between 06:00 and 22:00, while nocturnal blood pressure (nBP) represents the mean between 22:00 and 06:00. Clinic blood pressure: Clinic blood pressure measurements are taken between 08:00 and 12:00. After ensuring subjects have rested for at least 5 min, investigators use an electronic sphygmomanometer (YE670C, Jiangsu Yuyue Medical Equipment) to measure blood pressure on the non-dominant arm (left arm) and record the results. Home blood pressure: Home blood pressure measurements are performed daily, at 08:00–10:00 and 20:00–22:00, using a standard electronic sphygmomanometer (YE670C, Jiangsu Yuyue Medical Equipment).

### 2.8 Sample size

The total sample size for this study comprised 136 individuals, evenly distributed into a test group and a control group, with 68 participants in each. The primary objective of this research was to assess the recovery rate of blood pressure rhythm in both groups, both before and after treatment. Based on the preliminary examination of the participant cohort (Jiang et al., 2022), the dipper blood pressure recovery rate was observed to be 5/19 in the test group and 1/19 in the control group. Utilizing a 1:1 case ratio between the experimental and control groups, with a significance level of α = 0.05 (two-sided) and a power of β = 0.2, the required single-group sample size was calculated using PASS 11.0 software and determined to be 56 participants (N = 56). However, taking into account an anticipated dropout rate ranging from 15% to 20%, the final sample size was set at 136 individuals.

### 2.9 Safety assessment

Vital signs, including heart rate, body temperature, and respiratory rate, were monitored at each visit. Laboratory indicators such as blood routine tests (red blood cells, white blood cells, platelets, hemoglobin, neutrophils), blood biochemistry indexes (creatinine, albumin, alanine aminotransferase, and aspartate aminotransferase), and urine urination routine tests (red blood cells, white blood cells, urine protein, and urobilinogen) were checked pre- and post-medication administration.

### 2.10 Ethical considerations

The studies involving human participants were reviewed and approved by the Ethics Committee of the Institute of Basic Theory for Chinese Medicine, China Academy of Chinese Medical Sciences (Ethical Approval Number: 2020-KY-EC-011). The patients/participants provided their written informed consent to participate in this study. The investigational product used in this trial, Quanduzhong capsules, is a marketed traditional Chinese medicine approved by the National Medical Products Administration (NMPA) of China. Prior to its initial approval, comprehensive non-clinical toxicological and safety pharmacological studies were conducted in accordance with regulatory requirements, establishing its safety profile for human use.

### 2.11 Statistical methods

All research data were double-entered into the EpiData database and thoroughly tested for consistency. Statistical analysis was performed using SPSS 24.0 software. Measurement data that adhered to a normal distribution were expressed as mean ± SD, while categorical data were described as [frequency (percentage)]. A t-test was employed to compare measurement data between groups when the data conformed to a normal distribution. For data that did not conform to normal distribution, the nonparametric Wilcoxon rank-sum test was used. Ranked data were analyzed with the Cochran–Mantel–Haenszel test. All statistical tests were conducted with two-sided significance, and a *P-value* of less than 0.05 was considered statistically significant, indicating a difference between the groups.

Special data processing: missing data: For missing data of primary efficacy endpoints, the last observation carried forward (LOCF) method was applied for data imputation. Missing data for secondary efficacy endpoints were not carried forward. Outliers: data points determined to be outliers by investigators based on clinical judgment will be excluded after referring back to the source data to rule out data entry errors.

## 3 Results

### 3.1 Fingerprint spectrum of Quanduzhong capsules

**FIGURE 1:**
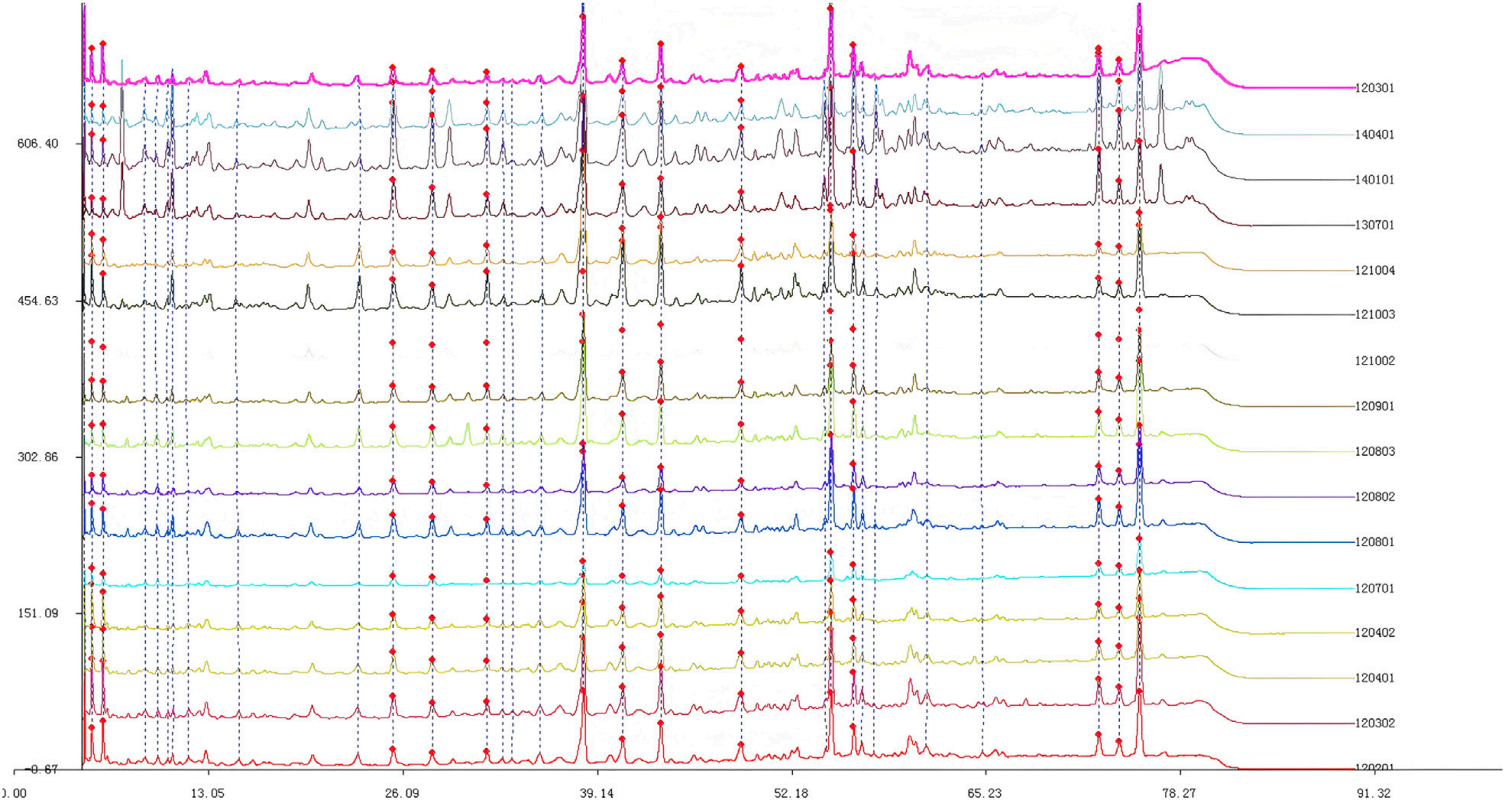
Fingerprint spectrum of 16 batches of Quanduzhong capsules.

**FIGURE 2:**
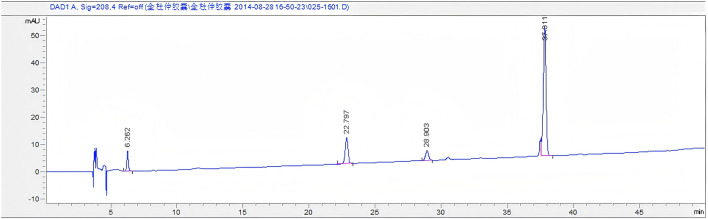
Mixed reference substances. From left to right: paeoniflorin, chlorogenic acid, loganin, and pinoresinol diglucoside.

The chromatogram of the mixed reference substances is depicted in Figure 1. Sample solutions derived from 16 distinct batches of Quanduzhong capsules were injected to produce the fingerprint spectrum, as illustrated in Figure 1. These chromatograms were subsequently imported into the Similarity Evaluation System 2004A, operated by the National Pharmacopoeia Commission. Utilizing the peaks delineated on the reference chromatogram (Figure 2) as reference points, multi-point adjustments were executed, and the similarity was calculated. The similarity scores of the fingerprint spectrum for all 16 batches of Quanduzhong capsules were all above 0.8 (0.806, 0.818, 0.883, 0.885, 0.897 0.936, 0.906, 0.886, 0.841, 0.825, 0.820, 0.839, 0.903, 0.909, 0.898, and 0.806) (Supplementary Table S1), signifying a high degree of commonality among the peaks and a consistent quality across different batches of the medicinal material.

### 3.2 LC-MS/MS analysis of Quanduzhong capsules

A total of 51 chromatographic peaks of Quanduzhong capsules were identified by LC‒MS/MS. The total ion chromatogram (TIC) and base peak chromatogram (BPC) are shown in Supplementary Figures S1,S2. Supplementary Table S2 shows the results of the chemical components in Quanduzhong capsules: they contain aucubin, chlorogenic acid, geniposide, terpineol diglucoside, and so on (Xu et al., 2023).

### 3.3 Patient enrollment

**FIGURE 3:**
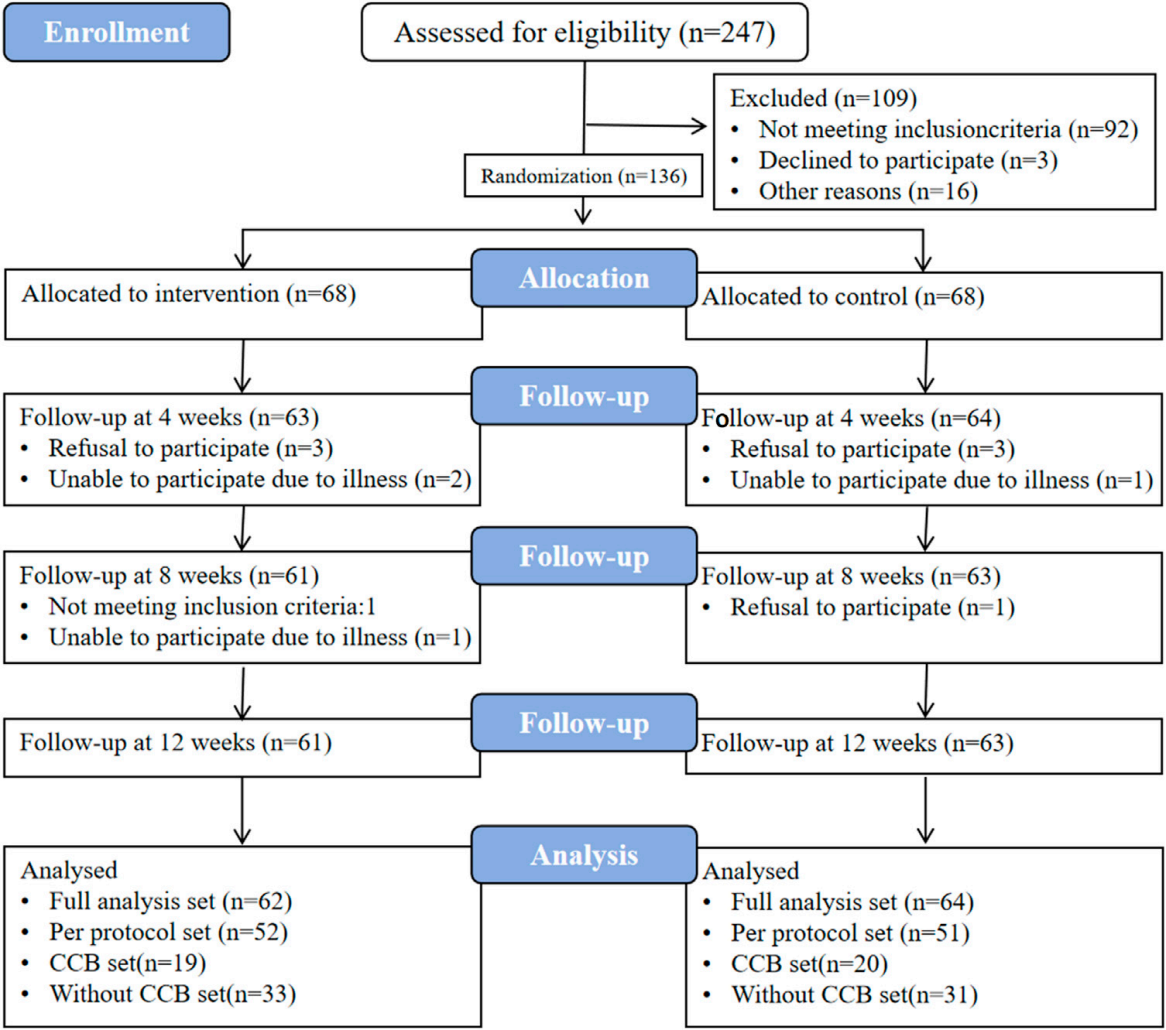
Case flow chart. FAS, full analysis set. PPS, per-protocol set. CCB, calcium channel blockers. The “CCB set” refers to treatment regimens involving concomitant use of CCB antihypertensive medications; the Without CCB set denotes combinations of other antihypertensive drug classes excluding CCBs, including beta-receptor blockers, angiotensin-converting enzyme inhibitors (ACEIs)/angiotensin receptor blockers (ARBs), and diuretics.

From 8/11/2021 to 10/2/2021, 136 participants were enrolled in the study, evenly distributed into 68 individuals in the test and control groups. Of these, 124 participants completed the study, with 61 in the test group and 63 in the control group. After thorough data cleaning and verification, the final dataset for per-protocol analysis consisted of 103 participants, comprising 52 from the test group and 51 from the control group (as depicted in Figure 3). For further details, please refer to the supplementary materials (Case shedding details).

### 3.4 Baseline clinical characteristics

Ultimately, the full analysis set (FAS) comprised 126 participants, with 62 individuals in the test group and 64 in the control group. The test group consisted of 28 men and 34 women, with a mean age of 60.02 ± 9.17 years, while the control group included 28 males and 36 females, with a mean age of 59.92 ± 8.83 years. For the per-protocol set (PPS), the study encompassed a total of 103 participants, with 52 individuals in the test group and 51 in the control group. The test group comprised 23 men and 29 women, having an average age of 59.79 ± 9.22 years, while the control group consisted of 22 men and 29 women, with an average age of 59.43 ± 8.77 years. There were no statistically significant differences between the two groups in either the FAS or PPS (*P* > 0.05). Upon comparing baseline characteristics such as weight, height, body mass index (BMI), history of smoking, drinking, drug allergies, food allergies, and other allergies, as well as hypertension grading and risk level grading, no statistically significant differences were observed between the two groups, indicating their comparability (Table 3). No significant differences in baseline were noted in the CCB and Without CCB sets (Supplementary Table S3).

**TABLE 3:** Comparison of general information between the two groups.

Characteristics	FAS		PPS	
	Test (n = 62)	Control (n = 64)	Test (n = 52)	Control (n = 51)
Source of cases (case/%)				
Outpatient	43 (69.40)	48 (75.00)	34 (65.38)	39 (76.47)
Hospitalization	19 (30.60)	16 (25.00)	18 (34.62)	12 (23.53)
Gender (case/%)				
Male	28 (45.20)	28 (43.80)	23 (44.23)	22 (43.14)
Female	34 (54.80)	36 (56.30)	29 (55.77)	29 (56.86)
Age	60.02 ± 9.17	59.92 ± 8.83	59.79 ± 9.22	59.43 ± 8.77
BMI	26.64 ± 3.23	25.81 ± 3.06	26.68 ± 3.14	25.67 ± 2.86
Weight	71.80 ± 14.35	69.16 ± 10.86	72.21 ± 14.26	69.54 ± 9.34
Height	163.52 ± 9.85	163.47 ± 8.83	163.88 ± 9.97	164.51 ± 7.7
Nationality (case/%)				
Han nationality	60 (96.80)	64 (100.00)	50 (96.15)	51 (100.00)
Others	2 (3.20)	0 (0.00)	2 (3.85)	0 (0.00)
Smoking history (case/%)				
No smoking	44 (71.00)	47 (73.40)	36 (69.23)	38 (74.51)
Occasional smoking (<10/day)	5 (8.10)	6 (9.40)	4 (7.69)	5 (9.80)
Frequent smoking (>10/day)	8 (12.90)	7 (10.90)	7 (13.46)	4 (7.84)
Smoking cessation	5 (8.10)	4 (6.30)	5 (9.62)	4 (7.84)
Drinking history (case/%)				
No drinking	42 (67.70)	39 (60.90)	34 (65.38)	31 (60.78)
Occasional drinking (<50 g/day)	13 (21.00)	18 (28.10)	11 (21.15)	15 (29.42)
Frequent drinking (>50 g/day)	6 (9.70)	4 (6.30)	6 (11.55)	2 (3.92)
Alcohol abstinence	1 (1.60)	3 (4.70)	1 (1.92)	3 (5.88)
Drug allergy history (case/%)				
Yes	6 (9.70)	3 (4.70)	6 (11.55)	3 (5.88)
No	56 (90.30)	61 (95.30)	46 (88.45)	48 (94.12)
History of other allergies (case/%)				
Yes	2 (3.20)	2 (3.10)	2 (3.85)	2 (3.92)
No	60 (96.80)	62 (96.90)	50 (96.15)	49 (96.08)
Hypertension grading (case/%)				
Grade 1	4 (6.50)	9 (14.10)	3 (5.77)	9 (17.65)
Grade 2	27 (43.50)	31 (48.40)	23 (44.23)	24 (47.06)
Grade 3	31 (50.00)	24 (37.50)	26 (50.00)	18 (35.29)
Risk level of hypertension (case/%)				
Low risk	4 (6.50)	6 (9.40)	3 (5.77)	6 (11.76)
Middle risk	18 (29.00)	20 (31.30)	14 (26.92)	15 (29.41)
High risk	7 (11.30)	7 (10.90)	5 (9.62)	5 (9.80)
Ultra high risk	33 (53.20)	31 (48.40)	30 (57.69)	25 (49.03)
Use of antihypertensive drugs (case)				
CCB	39	39	33	31
β-blocker	21	17	17	12
ACEI/ARB	35	36	27	29
Diuretics	5	6	3	2

FAS, full analysis set. PPS, per-protocol set. BMI: body mass index. CCB, calcium channel blockers; ACEI, angiotensin-converting enzyme inhibitors; ARB, angiotensin II receptor blockers.

### 3.5 Blood pressure SD

**FIGURE 4:**
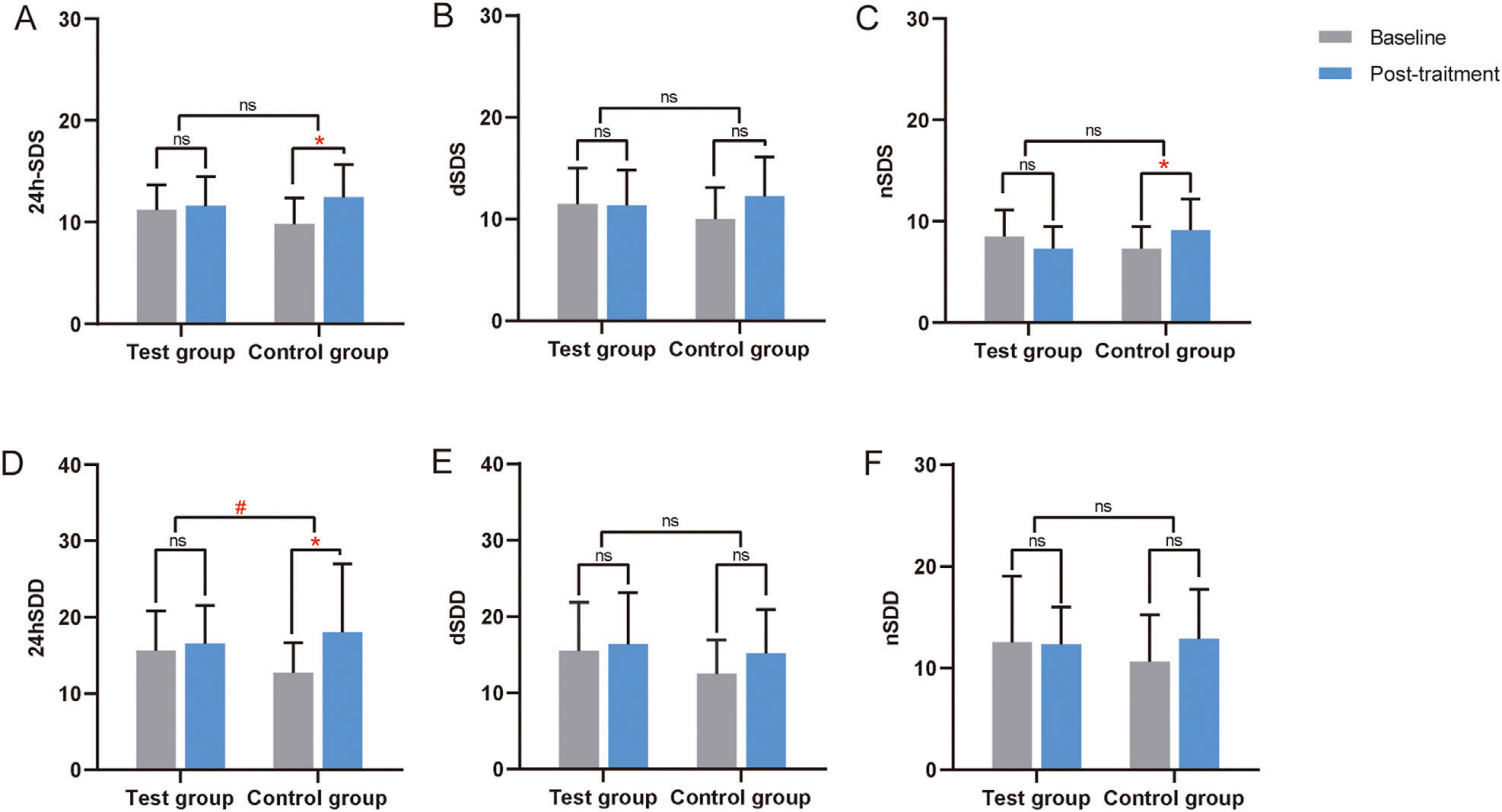
Changes in the SD from the ABPM of the Without CCB set. **(A)** Changes in 24-hour standard deviation of systolic blood pressure (24h-SDS) from the ABPM of the Without CCB set. **(B)** Changes in daytime standard deviation of systolic blood pressure (dSDS) from the ABPM of the Without CCB set. **(C)** Changes in nocturnal standard deviation of systolic blood pressure (nSDS) from the ABPM of the Without CCB set. **(D)** Changes in 24-hour standard deviation of diastolic blood pressure (24hSDD) from the ABPM of the Without CCB set. **(E)** Changes in daytime standard deviation of diastolic blood pressure (dSDD) from the ABPM of the Without CCB set. **(F)** Changes in nocturnal standard deviation of diastolic blood pressure (nSDD) from the ABPM of the Without CCB set. *, Compared with this group before treatment, *P < 0.05*. #, Compared the differences before and after treatment between the two groups, *P < 0.05*. CCB, calcium channel blocker. ns, non-significant.

No significant differences in blood pressure SD were found between the two groups in the FAS, PPS, and CCB sets (*P* > 0.05) (Supplementary Table S4; Table 4). In the Without CCB set, SD regulation in the test group was better than in the control group, with a statistically significant difference noted in the 24SDD (*P* < 0.05) (Table 4; Figure 4).

**TABLE 4:** Changes in blood pressure SD of CCB stratified analysis in PPS.

Items	CCB			Without CCB		
	Test (n = 33)	Control (n = 31)	*P*	Test (n = 19)	Control (n = 20)	*P*
24hSDS	1.39 ± 2.86^##^	2.15 ± 3.89^##^	0.85	0.44 ± 3.95	2.67 ± 4.27^#^	0.07
dSDS	1.22 ± 3.92	2.02 ± 4.05^#^	0.84	−0.17 ± 4.76	2.25 ± 5.31	0.10
nSDS	1.33 ± 4.25	0.6 ± 4.14	0.30	0.74 ± 4.05	1.87 ± 3.07^#^	0.22
24hSDD	2.33 ± 6.41	2.59 ± 6.39	0.76	0.97 ± 6.32	5.31 ± 9.07^#^	0.04*
dSDD	1.71 ± 7.04	1.84 ± 6.7	0.66	0.82 ± 8.25	2.67 ± 6.42	0.10
nSDD	1.93 ± 4.41^#^	1.63 ± 6.75	0.75	−0.19 ± 6.22	2.23 ± 6.19	0.21

Compared with this group before treatment, #, *P < 0.05*, ##, *P < 0.01*. Comparing the differences before and after treatment between the two groups, *P < 0.05. CCB, calcium channel blockers. 24h-SDS:24-hour standard deviation of systolic blood pressure; dSDS: daytime standard deviation of systolic blood pressure; nSDS: nocturnal standard deviation of systolic blood pressure; 24hSDD: 24-h standard deviation of diastolic blood pressure; dSDD: daytime standard deviation of diastolic blood pressure; nSDD: Nocturnal standard deviation of diastolic blood pressure. The CCB set refers to treatment regimens involving concomitant use of CCB antihypertensive medications; the Without CCB set denotes combinations of other antihypertensive drug classes excluding CCBs, including beta-receptor blockers, ACEI/ARBs, and diuretics.

### 3.6 Blood pressure CV

**FIGURE 5:**
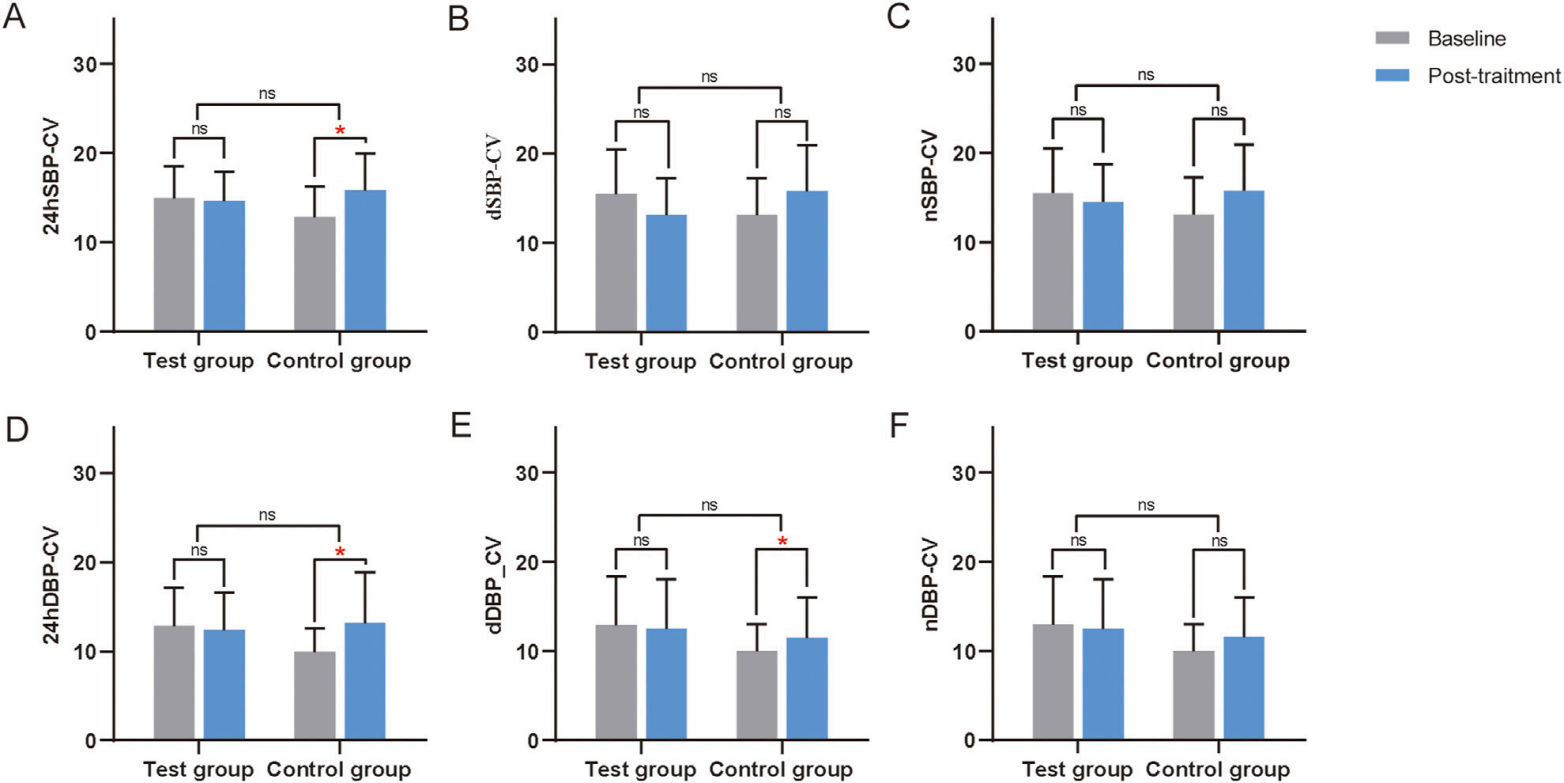
Changes in the CV from the ABPM of the Without CCB set. **(A)** Changes in 24-h coefficient of variation of systolic blood pressure (24hSBP-CV) from the ABPM of the Without CCB set. **(B)** Changes in daytime coefficient of variation of systolic blood pressure (dSBP-CV) from the ABPM of the Without CCB set. **(C)** Changes in nocturnal coefficient of variation of systolic blood pressure (nSBP-CV) from the ABPM of the Without CCB set. **(D)** Changes in the 24-hour coefficient of variation of diastolic blood pressure (24DBP-CV) from the ABPM of the Without CCB set. **(E)** Changes in daytime coefficient of variation of diastolic blood pressure (dDBP-CV) from the ABPM of the Without CCB set. **(F)** Changes in the nocturnal coefficient of variation of diastolic blood pressure (nDBP-CV) from the ABPM of the Without CCB set. ns, non-significant. *, Compared with this group before treatment, *P* < 0.05. CCB, calcium channel blockers. ns, non-significant.

In the FAS, PPS, CCB, and Without CCB sets, no significant differences in blood pressure CV were found between the two groups (*P > 0.05*) (Supplementary Table S1; Table 5). However, in the Without CCB set, all CV metrics in the test group showed improvement compared to the control group (Table 5; Figure 5).

**TABLE 5:** Changes in blood pressure CV of CCB stratified analysis in the PPS.

Items	CCB			Without CCB		
	Test (n = 33)	Control (n = 31)	*P*	Test (n = 19)	Control (n = 20)	*P*
24hSBP-CV	1.89 ± 3.76^##^	2.26 ± 5.93^#^	0.72	−0.3 ± 5.32	3.02 ± 5.63^#^	0.10
dSBP-CV	1.34 ± 4.42	2.23 ± 6.24	0.43	−0.99 ± 6.54	2.66 ± 7.01	0.14
nSBP-CV	1.56 ± 5.58	0.1 ± 5.62	0.49	−0.01 ± 5.53	1.89 ± 4^#^	0.33
24DBP-CV	1.87 ± 5.48	1.87 ± 4.3^#^	0.88	−0.47 ± 5.47	3.21 ± 5.97	0.07
dDBP-CV	1.68 ± 6.24	1.02 ± 5.52	0.94	−0.44 ± 7.05	1.56 ± 4.58	0.20
nDBP-CV	1.14 ± 3.01^#^	0.98 ± 4.43	0.83	−1.39 ± 5.22	0.62 ± 4.57	0.23

Compared with this group before treatment, #, *P* < 0.05, ##, *P* < 0.01. CCB, calcium channel blockers. 24hSBP-CV: 24-h coefficient of variation of systolic blood pressure; dSBP-CV: daytime coefficient of variation of systolic blood pressure; nSBP-CV: nocturnal coefficient of variation of systolic blood pressure; 24DBP-CV: 24-h coefficient of variation of diastolic blood pressure; dDBP-CV: daytime coefficient of variation of diastolic blood pressure; nDBP-CV: Nocturnal coefficient of variation of diastolic blood pressure. The CCB set refers to treatment regimens involving concomitant use of CCB antihypertensive medications; the Without CCB set denotes combinations of other antihypertensive drug classes excluding CCBs, including beta-receptor blockers, ACEI/ARBs, and diuretics.

### 3.7 Recovery rate of dipper blood pressure rhythm

There were statistically significant differences in the recovery rate of dipper blood pressure rhythm post-medication in four sets, compared to baseline, for both the test and control groups (*P < 0.05*), but no statistically significant differences were observed between the two groups after treatment (Supplementary Tables S6, S7).

### 3.8 Mean ABPM blood pressure

In the FAS and PPS, blood pressure decreased to varying degrees in both groups after treatment, and the nocturnal blood pressure exhibited a significant decline (*P <* 0.05). However, the difference between the test group and control group was not statistically significant (*P* > 0.05) (Supplementary Table S8). In the CCB set, the antihypertensive effect in the control group was significantly better than that in the test group, as were the mean value of 24-h diastolic blood pressure (24hMBP), the mean value of daytime diastolic blood pressure (dMBP), the mean value of nocturnal diastolic blood pressure (nMBP), with a statistically significant difference (*P* < 0.05). In the Without CCB set, the antihypertensive effect in the test group was significantly better than that in the control group, but the difference was not statistically significant (*P >* 0.05) (Supplementary Table S9).

### 3.9 Other secondary efficacy indexes

There were no statistically significant differences between the text and control groups with respect to clinic blood pressure, home blood pressure, blood pressure control efficacy, and antihypertensive effect (*P* > 0.05).

### 3.10 Safety

In terms of safety parameters such as body temperature, respiration, and heart rate, no statistically significant differences were found at baseline, and after 4 weeks, 8 weeks, and 12 weeks of medication (*P* > 0.05). Similarly, no significant differences were noted in test indices like alanine transaminase, aspartate transaminase, and creatinine at baseline and after 12 weeks of medication (*P* > 0.05) (Supplementary Tables S10, S11).

Adverse event report: A total of 13 adverse events were recorded during the study period (Supplementary Table S12), including one serious adverse event (SAE): a subject experienced a sudden acute myocardial infarction, was hospitalized, and subsequently died. The subject belonged to the control group and had not taken the investigational drug; therefore, the event was determined to be unrelated to the trial medication. The remaining 12 cases were non-serious adverse events. Among these, three subjects exhibited hypotensive reactions following the administration of the investigational drug. These symptoms resolved after discontinuation of Total Eucommia Capsules, and all three subjects requested to withdraw from the clinical trial.

## 4 Discussion

The study of blood pressure variability (BPV) dates to the 1980s (Floras et al., 1981), when the initial recordings of blood pressure fluctuations emerged. These measurements revealed that blood pressure values are not static, but instead exhibit variability, thus introducing the concept of “BPV.” Over time, with advancements in blood pressure monitoring, scholars recognized a regular pattern of blood pressure fluctuations over specific time periods, particularly across a 24-hour cycle. This led to the development of the concept of “blood pressure circadian rhythm”. Typically, a normal blood pressure circadian rhythm exhibits a “double peak and a valley” curve, characterized by a 10%–20% drop in systolic blood pressure during sleep compared to waking hours (Gumz et al., 2023). This pattern has been aptly named “dipper” blood pressure due to its resemblance to a long ladle.

Although the concept of blood pressure circadian rhythm is not found in ancient Chinese medicine, numerous references to “rhythm” are present. Through comprehensive studies of ancient literature, contemporary practitioners of traditional Chinese medicine (TCM) have established Chinese Medicine Time Medicine (Zheng et al., 2024). This discipline embraces the concepts of circadian rhythms, including the mutual growth and decline of Yin and Yang, the rhythm of Qi dynamics’ floating and sinking, the flow of defensive energy, the timing of the five visceral functions, and the influx of blood along meridians.

Extensive research indicates that BPV is positively associated with the onset, progression, and severity of cardiac (Yang et al., 2019), vascular (Sun et al., 2023), and renal damage (Zhang et al., 2021), as well as an elevated risk of cardiovascular events and mortality (Yang et al., 2019). The standard deviation (SD) and coefficient of variation (CV) of blood pressure are key metrics for assessing its volatility. Specifically, the SD and CV of 24-h, daytime, and nighttime blood pressure averages are utilized as variability indicators (Narita et al., 2023), providing insights into the dispersion of blood pressure monitoring values across different time periods and reflecting the extent of blood pressure fluctuations. However, normal reference values for these measurements have not yet been established. Notably, blood pressure rhythm abnormalities are typically observed in non-dippers and reverse-dippers (Staessen et al., 2000), and a higher SD and CV generally suggest greater BPV.

In recent years, TCM practitioners have successfully employed traditional Chinese medicine (Zhong et al., 2011; Zhang et al., 2025) and various TCM-appropriate techniques (Chen et al., 2023; Chen et al., 2024) to treat blood pressure circadian rhythm abnormalities, achieving promising clinical outcomes. Zhao and Zhang (2025) investigated the significant effect of Si Ni Tang (a traditional Chinese medicine) combined with Western medicine in managing hypertension, positively influencing patients’ BPV. Xu et al. (2023) observed 60 patients with Grade 1 essential hypertension, finding that diastolic blood pressure CV improved in the treatment group after 4 weeks of Quanduzhong capsules, with statistically significant differences compared to the control group (*P < 0.05*). In the current study, both subject groups exhibited an increase in blood pressure SD and CV, albeit the test group displayed a more modest rise than the control group. All patients were enrolled between 11 August and 2 October 2021 (summer season). With a 3-month visit schedule, all subjects completed the study by mid-November 2021 onward (winter season). Given the established seasonal variability of blood pressure—where higher SD and CV values are typically observed in winter versus summer (Narita et al., 2022; Barbosa et al., 2023; Du et al., 2023)—combined with our data suggesting a potential association between Quanduzhong capsule administration and apparent reductions in blood pressure SD/CV fluctuations, these trends appear broadly aligned with preliminary observations in related studies.

Blood pressure values have a positive correlation with blood pressure SD and CV (Parati et al., 2023), making them crucial indicators in evaluating BPV. Both subject groups showed a hypotensive effect after medication, though the control group exhibited a more significant decrease in blood pressure. The antihypertensive properties of Duzhong can be broadly classified as being due to lignans, phenylpropanoids, cycloidal terpenoids, and flavonoids. These components exert antihypertensive effects through various mechanisms, including promoting nitric oxide release, inhibiting calcium influx, phosphodiesterase, and angiotensin activity, and blocking endothelin (Shi et al., 2022), thereby providing bidirectional regulation of blood pressure (Zhang et al., 2023). Several clinical studies (Yang et al., 2021; Xu et al., 2023; Wang et al., 2025) have demonstrated the effectiveness of single-drug preparations of Duzhong, such as Duzhong granules and Quanduzhong capsules. However, these findings contrast with the results of the current study. This discrepancy may be attributed to the potential ceiling effect of the medication. The current study was an add-on clinical trial, with subjects who had regularly used Western antihypertensive medication before enrollment, bringing their blood pressure close to or within standard limits. This may have reached the maximum drug effect in the human body, resulting in an insignificant antihypertensive effect after Quanduzhong capsules were administered.

During data analysis, the project team conducted a stratified analysis based on the subjects’ original Western antihypertensive medication regimen, revealing a significantly higher blood pressure drop in the test group than the control group among subjects not using calcium antagonists. Conversely, there were no significant differences in blood pressure drop between the two groups using calcium antagonists, further supporting the possibility of a drug ceiling effect. Meanwhile, we observed that in patients not concurrently using CCBs, the blood pressure rhythm of those in the Quanduzhong capsule group showed significant improvement, particularly evident in the 24hSDD (*P* < 0.05). This may be related to the competitive antagonism between *E. ulmoides* and CCB. Quercetin is one of the primary active metabolites in *E. ulmoides* responsible for its antihypertensive effects (Huang et al., 2021). It exerts a dual inhibitory effect on both voltage-dependent calcium channels and receptor-operated calcium channels in vascular smooth muscle cells, thereby reducing intracellular free calcium levels. This leads to vasodilation and a subsequent reduction in blood pressure (He et al., 2014).

This study produced some encouraging results, but after 12 weeks of treatment, in the FAS and PPS sets, the results showed no statistically significant differences between the treatment group and the control group in SD and CV. There were also some limitations. The impact of the novel coronavirus pandemic resulted in a higher dropout rate than anticipated. Additionally, the study did not take into account the seasonal variability of blood pressure. In future studies, we aim to increase the sample size, conduct cohort studies with longer follow-ups, and carry out multicenter, large-sample clinical trials to better understand the practical efficacy of Quanduzhong capsules in regulating the circadian rhythm of blood pressure.

## 5 Conclusion

Quanduzhong capsules possess a potential regulatory effect on the circadian rhythm of blood pressure, with a more pronounced therapeutic efficacy when not combined with CCB.

## Data Availability

The original contributions presented in the study are included in the article/Supplementary Material; further inquiries can be directed to the corresponding authors.

## Glossary

24 DBP-CV: 24-hour coefficient of variation of diastolic blood pressure
24 h SBP-CV: 24-hour coefficient of variation of systolic blood pressure
24 h SDD: 24-hour standard deviation of diastolic blood pressure
24 h-SDS: 24-hour standard deviation of systolic blood pressure
ABPM: ambulatory blood pressure monitoring
BMI: body mass index
BPC: base peak chromatogram
BPV: blood pressure variability
CV: coefficient of variation
dBP: daytime blood pressure
DBP: diastolic blood pressure
dDBP-CV: daytime coefficient of variation of diastolic blood pressure
dSBP-CV: daytime coefficient of variation of systolic blood pressure
dSDD: daytime standard deviation of diastolic blood pressure
dSDS: daytime standard deviation of systolic blood pressure
ESI: electrospray ionization
FAS: full analysis set
GAP: good agricultural practice
LOCF: last observation carried forward
MS: mass spectrometry
nBP: nocturnal blood pressure.
nDBP-CV: nocturnal coefficient of variation of diastolic blood pressure
nSBP-CV: nocturnal coefficient of variation of systolic blood pressure
nSDD: nocturnal standard deviation of diastolic blood pressure
nSDS: nocturnal standard deviation of systolic blood pressure
PPS: per-protocol set
SBP: systolic blood pressure
SD: standard deviation
TCM: traditional Chinese medicine
TIC: total ion chromatogram
UPLC-Q-TOF: ultra-performance liquid chromatography quadrupole time-of-flight
