# Supplementary material for: Efficacy of Quanduzhong capsules in regulating blood pressure circadian rhythm in essential hypertension: a multicenter, randomized, add-on controlled clinical trial
Source: Front Pharmacol. 2025 Oct 8;16:1659073. doi: 10.3389/fphar.2025.1659073 (PMC12540364; doi:10.3389/fphar.2025.1659073)
Supplement: Supplementary file 1 [file DataSheet1.docx]

**SUPPLEMENTARY MATERIALS**

**Case shedding details**

A total of 136 participants were enrolled in the study, comprising 68 individuals in both the test and control groups. As the study progressed, 7 participants (3 from the test group and 4 from the control group) discontinued their follow-up, 3 participants (from the test group) requested withdrawal due to adverse events, 1 individual (from the control group) passed away, and 1 participant (from the test group) was excluded upon the investigator's determination that this individual did not meet the "non-dipper blood pressure rhythm" inclusion criterion. In sum, 124 participants completed the study, with the test group consisting of 61 individuals and the control group comprising 63.

Upon creating the database, data were cleaned and verified. In alignment with the principle of excluding data from the full analysis set, 3 participants (from the test group) who did not meet the crucial "non-dipper blood pressure rhythm" inclusion criterion, 1 individual (from the control group) exceeding the age limit for inclusion by more than 5 years, 5 participants (2 from the test group and 3 from the control group) who failed the ambulatory blood pressure examination report, 1 individual with pancreatic cancer and liver metastasis (from the test group), and 1 participant (from the control group) who passed away due to acute myocardial infarction three days post-enrollment were all omitted. Consequently, 126 participants (62 in the test group and 64 in the control group) were included in the full analysis set. From this full analysis set, 10 participants who did not complete the entire test procedure (5 each from the experimental and control groups) and 13 participants (5 from the test group and 8 from the control group) with a window period exceeding 7 days were excluded. Ultimately, 103 participants (52 from the test group and 51 from the control group) were included in the per protocol analysis set. Stratified by whether CCB was used, 33 people in the experimental group used CCB, and 19 people did not use CCB; 31 people in the control group used CCB, and 20 people did not use CCB.

**Table S1 Similarity of Fingerprint Profiles for 16 Batches of Samples Quanduzhong capsule**

| **Batch** | 120201 | 120302 | 120401 | 120402 | 120701 | 120801 | 120802 | 120803 |
| --- | --- | --- | --- | --- | --- | --- | --- | --- |
| **Similarity** | 0.806 | 0.818 | 0.883 | 0.885 | 0.897 | 0.936 | 0.906 | 0.886 |
| **Batch** | 120901 | 121002 | 121003 | 121004 | 130701 | 140101 | 140401 | 120301 |
| **Similarity** | 0.841 | 0.825 | 0.820 | 0.839 | 0.903 | 0.909 | 0.898 | 0.806 |

**Table S2 Identification of chemical constituents in Quanduzhong capsule**

| **S.No** | **tR (min)** | **[M-H]^-^** | **Molecular formula** | **MS/MS fragments** | **Identification** |
| --- | --- | --- | --- | --- | --- |
| 1 | 7.20 | 387.1440 | C_21_H_24_O_7_ | 357.0973,341.2002,313.1083,151.0407 | Medioresinol |
| 2 | 6.93 | 549.1967 | C_27_H_34_O_12_ | 387.1446,372.1213,181.0519 | Medioresinol 4’-O-β-D-glucopyranoside and its isomer |
| 3 | 7.20 | 549.1970 | C_27_H_34_O_12_ | 503.2497,387.1447,372.1211,151.0418 |  |
| 4 | 5.77 | 711.2499 | C_33_H_44_O_17_ | 549.1981,550.2030,387.1457 | (+)-Medioresinol Di-O-β-D-glucopyranoside |
| 5 | 6.98 | 357.1341 | C_20_H_22_O_6_ | 342.1097,151.0413,136.0179 | Pinoresinol and its isomer |
| 6 | 10.44 | 357.1335 | C_20_H_22_O_6_ | 327.1143,324.0899,311.1492,281.0730,151.0406,136.0178 |  |
| 7 | 11.72 | 357.1340 | C_20_H_22_O_6_ | 313.2380,311.0935,252.0813,201.1154 |  |
| 8 | 6.98 | 519.1859 | C_26_H_32_O_11_ | 357.1340,151.0412 | (+)-pinoresinol-β-D-glucoside |
| 9 | 5.57 | 681.2392 | C_32_H_42_O_16_ | 519.1855,357.1339 | Pinoresinol diglucoside |
| 10 | 7.89 | 373.1287 | C_20_H_22_O_7_ | 358.1055,343.1185,325.1083,313.1079,298.0851,108.0241 | 1-Hydroxypinoresinol and its isomer |
| 11 | 9.14 | 373.1286 | C_20_H_22_O_7_ | 355.1180,327.2135,285.2044,235.0626,201.1137,179.0717,164.0488,123.0489 |  |
| 12 | 10.42 | 373.1281 | C_20_H_22_O_7_ | 358.1049,327.2176,203.0719,151.0406 |  |
| 13 | 12.58 | 373.1287 | C_20_H_22_O_7_ | 358.1058,327.2170,167.0355,108.0233 |  |
| 14 | 6.15 | 535.1815 | C_26_H_32_O_12_ | 373.1294,343.1191,313.1086 | (+)-1-hydroxypinoresinol 4’’-O-β-D-glucopyranoside and its isomer |
| 15 | 8.65 | 535.1813 | C_26_H_32_O_12_ | 373.1295,358.1050 |  |
| 16 | 5.06 | 697.2343 | C_32_H_42_O_17_ | 651.2329,535.1781,373.1291,343.1186,211.0625 | 1-hydroxypinoresinol 4’,4’’-O-β-D-glucopyranoside |
| 17 | 7.13 | 417.1552 | C_22_H_26_O_8_ | 402.1317,387.1084,359.1154,181.0516,166.0279 | Syringaresinol |
| 18 | 5.94 | 579.2073 | C_28_H_36_O_13_ | 417.1551,402.1348,181.0518 | Syringaresinol-O-β-D-glucopyranoside and its isomer |
| 19 | 7.13 | 579.2074 | C_28_H_36_O_13_ | 417.1549,402.1319,181.0517 |  |
| 20 | 5.94 | 741.2605 | C_34_H_46_O_18_ | 579.2071,417.1553 | Liriodendrin and its isomer |
| 21 | 6.93 | 741.2606 | C_34_H_46_O_18_ | 519.1889,417.1534,357.1328,323.0966,221.0464,161.0456 |  |
| 22 | 5.71 | 375.1445 | C_20_H_24_O_7_ | 360.1207,327.1243,297.0744,257.0817,241.0514,189.0552,108.0231 | Olivil |
| 23 | 6.53 | 375.1446 | C_20_H_24_O_7_ | 360.1221,327.1248,195.0659,191.0717,179.0714,122.0387 | Cycloolivil |
| 24 | 2.53 | 345.1186 | C_15_H_22_O_9_ | 299.1119,161.0300,119.0340 | Aucubin |
| 25 | 13.63 | 507.1655 | C_21_H_32_O_14_ | 491.1377 | b-D-Glucopyranoside,(1S,4aR,5S,7aS)-1,4a,5,7a-tetrahydro-5-hydroxy-7-(hydroxymethyl)cyclopenta[c]pyran-1-yl |
| 26 | 7.55 | 187.0983 | C_9_H_16_O_4_ | 169.0880,125.0993 | Eucommiol |
| 27 | 10.09 | 171.1033 | C_9_H_16_O_3_ | 153.0938,127.1135,99.0843 | Deoxyeucommiol and its isomer |
| 28 | 11.34 | 171.1034 | C_9_H_16_O_3_ | 153.0922,127.1129,125.0982,99.0835 |  |
| 29 | 4.43 | 179.0352 | C_9_H_8_O_4_ | 135.0465 | Caffeic acid and its isomers |
| 30 | 10.29 | 179.0351 | C_9_H_8_O_4_ | 134.9897,105.0386,91.0000 |  |
| 31 | 6.22 | 179.0356 | C_9_H_8_O_4_ | 134.9902,109.0312 |  |
| 32 | 5.3 | 179.0355 | C_9_H_8_O_4_ | 135.0466 |  |
| 33 | 9.99 | 207.0665 | C_11_H_12_O_4_ | 179.0356 | Ethyl 3-(3,4-dihydroxyphenyl)acrylate and its isomer |
| 34 | 10.28 | 207.0666 | C_11_H_12_O_4_ | 179.0355,135.0464 |  |
| 35 | 6.37 | 193.0510 | C_10_H_10_O_4_ | 178.0290 | Pinusolidic acid and its isomer |
| 36 | 7.92 | 193.0507 | C_10_H_10_O_4_ | 178.0270 |  |
| 37 | 8.42 | 193.0510 | C_10_H_10_O_4_ | 178.0279,136.0180,149.0628 |  |
| 38 | 7.97 | 181.0508 | C_9_H_10_O_4_ | 108.0241 | 3,4-dihydroxyphenylpropionic acid |
| 39 | 3.54 | 353.0873 | C_16_H_18_O_9_ | 191.0569,173.0460 | Chlorogenic acid |
| 40 | 8.63 | 167.0388 | C_8_H_8_O_4_ | 96.0627 | Vanillic acid and its isomer |
| 41 | 4.49 | 167.0356 | C_8_H_8_O_4_ | 152.0131,123.0427,108.0243,91.0219 |  |
| 42 | 15.03 | 163.0407 | C_9_H_8_O_3_ | 229.1065,119.0527 | p-Coumaric acid and its isomer |
| 43 | 5.84 | 163.0404 | C_9_H_8_O_3_ | 119.0520,93.0370 |  |
| 44 | 9.36 | 285.0403 | C_15_H_10_O_6_ | 151.0051,133.0313 | Kaempferol |
| 45 | 6.49 | 593.1503 | C_27_H_30_O_15_ | 285.0413 | Kaempferol-3-rutinoside |
| 46 | 9.4 | 301.0353 | C_15_H_10_O_7_ | 178.9996,151.0043 | Quercetin |
| 47 | 18.5 | 289.0716 | C_15_H_14_O_6_ | 289.0724,177.0548 | Catechin |
| 48 | 6.19 | 609.1461 | C_27_H_30_O_16_ | 301.0352 | Rutinum |
| 49 | 6.5 | 447.0925 | C_21_H_20_O_11_ | 285.0409 | Quercitrin |
| 50 | 7.55 | 367.2115 | C_22_H_28_N_2_O_3_ | 349.2026,331.1922,305.2127 | Hirsutine |
| 51 | 24.21 | 455.3518 | C_30_H_48_O_3_ |  | Betulinic acid |

Table S3 Comparison of general information between the two groups

| **Characteristics** | **CCB** | | **WITHOUT CCB** | |
| --- | --- | --- | --- | --- |
|  | **Test (n=33)** | **Control (n=31)** | **Test (n=19)** | **Control (n=20)** |
| **Source of cases** (case/%) |  |  |  |  |
| Outpatient | 17 (51.52)* | 25 (80.65)* | 17 (89.47) | 14 (70) |
| Hospitalization | 16 (48.48)* | 6 (19.35)* | 2 (10.53) | 6 (30) |
| **Gender** (case/%) |  |  |  |  |
| Male | 14 (42.42) | 14 (45.16) | 9 (47.37) | 8 (40) |
| Female | 19 (57.58) | 17 (54.84) | 10 (52.63) | 12 (60) |
| **Age** | 60.97±8.92 | 60.42±9.05 | 57.74±9.60 | 57.9±8.30 |
| **BMI** | 26.42±3.00 | 25.76±2.89 | 27.14±3.39 | 25.53±2.87 |
| **Weight** | 70.44±12.47 | 70.52±9.83 | 75.29±16.85 | 68.03±8.53 |
| **Height** | 162 (155,171) | 168 (160,171) | 165.58±9.32 | 163.25±7.13 |
| **Nationality** (case/%) |  |  |  |  |
| The han nationality | 31 (93.94) | 31 (100) | 19 (100) | 20 (100) |
| Others | 2 (6.06) | 0 (0) | 0 (0) | 0 (0) |
| **Smoking history** (case/%) |  |  |  |  |
| No smoking | 23 (69.7) | 23 (74.19) | 13 (68.42) | 15 (75) |
| Occasional smoking (＜10/day) | 2 (6.06) | 4 (12.9) | 2 (10.53) | 1 (5) |
| Frequent smoking (＞10/day) | 4 (12.12) | 3 (9.68) | 3 (15.79) | 1 (5) |
| Smoking cessation | 4 (12.12) | 1 (3.23) | 1 (5.26) | 3 (15) |
| **Drinking history** (case/%) |  |  |  |  |
| No drinking | 23 (69.7) | 17 (54.84) | 13 (68.42) | 15 (75) |
| Occasional drinking (＜50g/day) | 6 (18.18) | 11 (35.48) | 2 (10.53) | 1 (5) |
| Frequent drinking (＞50g/day) | 3 (9.09) | 1 (3.23) | 3 (15.79) | 1 (5) |
| Alcohol abstinence | 1 (3.03) | 2 (6.45) | 1 (5.26) | 3 (15) |
| **Drug allergy history** (case/%) |  |  |  |  |
| Yes | 28 (84.85) | 30 (96.77) | 18 (94.74) | 18 (90) |
| No | 5 (15.15) | 1 (3.23) | 1 (5.26) | 2 (10) |
| **History of other allergies** (case/%) |  |  |  |  |
| Yes | 32 (96.97) | 29 (93.55) | 18 (94.74) | 20 (100) |
| No | 1 (3.03) | 2 (6.45) | 1 (5.26) | 0 (0) |

CCB, Calcium Channel Blockers. BMI: Body Mass Index. The "CCB set" refers to treatment regimens involving concomitant use of CCB antihypertensive medications, the "without CCB set" denotes combinations of other antihypertensive drug classes excluding CCBs, including Beta-receptor blockers, ACEI/ARB, and diuretics.

**Table S4 Changes in blood pressure SD(FAS and PPS)**

| **Items** | **FAS difference value** | | | **PPS difference value** | | |
| --- | --- | --- | --- | --- | --- | --- |
|  | **Test (n=62)** | **Control (n=64)** | ***P*** | **Test (n=52)** | **Control (n=51)** | ***P*** |
| 24hSDS | 1.72±5.13^#^ | 2.29±5.37^##^ | 0.35 | 1.49±5.34 | 2.56±5.77^##^ | 0.33 |
| dSDS | 1.17±5.79 | 2.49±6.72^##^ | 0.24 | 0.87±6.04 | 2.40±6.49^#^ | 0.22 |
| nSDS | 1.16±5.33 | 1.02±4.75 | 0.87 | 0.99±5.56 | 0.80±5.08 | 0.86 |
| 24hSDD | 0.58(-1.61,3.4) | 0.8(-0.85,3.94)^##^ | 0.55 | 1.01±5.54 | 2.00±4.17^##^ | 0.29 |
| dSDD | 0(-2.5,4.01) | 0.53(-1.97,4.09) | 0.62 | 0.90±6.56 | 1.23±5.14 | 0.44 |
| nSDD | 0.16(-1.47,2.86) | 0.88(-1.24,3.31)^#^ | 0.72 | 0.22±4.10 | 0.84±4.45 | 0.71 |

Compared with this group before treatment, #, *P* < 0.05, ##, *P* < 0.01. FAS, full analysis set. PPS, per-protocol set. 24h-SDS,24-hour standard deviation of systolic blood pressure. dSDS,Daytime standard deviation of systolic blood pressure. nSDS, Noctural standard deviation of systolic blood pressure. 24hSDD, 24-hour standard deviation of diastolic blood pressure. dSDD, Daytime standard deviation of diastolic blood pressure. nSDD, Noctural standard deviation of diastolic blood pressure.

**Table S5 Changes in blood pressure CV(FAS and PPS)**

| **Items** | **FAS difference value** | | | **PPS difference value** | | |
| --- | --- | --- | --- | --- | --- | --- |
|  | **Test (n=62)** | **Control (n=64)** | ***P*** | **Test (n=52)** | **Control (n=51)** | ***P*** |
| 24hSBP-CV | 1.41±3.66^##^ | 2.01±3.86^##^ | 0.28 | 1.31±3.81^#^ | 2.35±4.01^##^ | 0.18 |
| dSBP-CV | 0.87±4.07 | 1.99±4.72^##^ | 0.15 | 0.71±4.25 | 2.11±4.54^##^ | 0.14 |
| nSBP-CV | 1.19±3.96^#^ | 1.18±3.51^##^ | 0.99 | 1.11±4.15 | 1.10±3.78^#^ | 0.99 |
| 24DBP-CV | 1.33(-1.12,4.25)^#^ | 1.42(-1.03,5.62)^##^ | 0.58 | 1.62(-2.38,5.30)^#^ | 1.89(-0.62,6.49)^##^ | 0.27 |
| dDBP-CV | 0.65(-2.39,4.36) | 1.26(-2.53,5.85) | 0.57 | 1.19(-2.65,5.03) | 2.01(-1.92,6.11)^#^ | 0.34 |
| nDBP-CV | 1.46±5.02^#^ | 2.01±6.11^#^ | 0.67 | 1.15±5.19 | 1.86±6.48^#^ | 0.54 |

Compared with this group before treatment, #, *P* < 0.05, ##, *P* < 0.01. 24hSBP-CV, 24-hour coefficient of variation of systolic blood pressure. dSBP-CV, Daytime coefficient of variation of systolic blood pressure. nSBP-CV, Noctural coefficient of variation of systolic blood pressure. 24DBP-CV, 24-hour coefficient of variation of diastolic blood pressure. dDBP-CV, Daytime coefficient of variation of diastolic blood pressure. nDBP-CV, Noctural coefficient of variation of diastolic blood pressure.

**Table S6** **Percentage of patients with circadian restoration(FAS and PPS)**

| **Items** | **Type of blood pressure rhythm** | **FAS** | | | **PPS** | | |
| --- | --- | --- | --- | --- | --- | --- | --- |
|  |  | **Test (n=62)** | **Control (n=64)** | ***P*** | **Test (n=52)** | **Control (n=51)** | ***P*** |
| Baseline | Dipper | 0 (0) | 0 (0) |  | 0 (0) | 0 (0) |  |
|  | Non-dipper | 62 (100) | 64 (100) |  | 52 (100) | 51 (100) |  |
| Post-treatment | Dipper | 17 (27.40)^##^ | 14 (21.90)^##^ |  | 15 (28.80)^##^ | 13 (25.50)^##^ |  |
|  | Non-dipper | 45 (72.60) | 50 (78.10) | 0.47 | 37 (71.20) | 38 (74.50) | 0.70 |

Compared with this group before treatment, ##, *P* < 0.01. FAS, full analysis set. PPS, per-protocol set.

**Table S7 Percentage of patients with circadian restoration of CCB stratified analysis in PPS**

| **Items** | **Type of blood pressure rhythm** | **CCB** | | | **WITHOUT CCB** | | |
| --- | --- | --- | --- | --- | --- | --- | --- |
|  |  | **Test (n=33)** | **Control (n=31)** | ***P*** | **Test (n=19)** | **Control (n=20)** | ***P*** |
| Baseline | Dipper | 0 (0) | 0 (0) |  | 0 (0) | 0 (0) |  |
|  | Non-dipper | 33 (100) | 31 (100) |  | 19 (100) | 20 (100) |  |
| Post-treatment | Dipper | 10 (30.30)^##^ | 7 (22.60)^##^ |  | 5 (26.30)^#^ | 6 (30.00)^##^ |  |
|  | Non-dipper | 23 (69.70) | 24 (77.40) | 0.49 | 14 (73.70) | 14 (70.00) | 0.80 |

Compared with this group before treatment, #, *P* < 0.05, ##, *P* < 0.01. CCB, Calcium Channel Blockers.

**Table S8 Changes in ABPM blood pressure(FAS and PPS)**

| **Items** | **FAS** **difference value** | | | **PPS difference value** | | |
| --- | --- | --- | --- | --- | --- | --- |
|  | **Test (n=62)** | **Control (n=64)** | ***P*** | **Test (n=52)** | **Control (n=51)** | ***P*** |
| 24hSBP | -1.24±9.73 | -2.29±14.21 | 0.80 | -2.12±10.00 | -5.02±17.02^#^ | 0.30 |
| dSBP | 0.42±9.96 | -1.73±15.99 | 0.56 | -0.38±10.15 | -3.53±17.05 | 0.26 |
| nSBP | -4.35±12.05^##^ | -6.86±18.15^##^ | 0.41 | -5.00±12.65^##^ | -8.51±19.03^##^ | 0.27 |
| 24hDBP | -1.23±8.61 | -2.70±8.13^#^ | 0.34 | -3.35±10.76^#^ | -4.12±8.18^##^ | 0.22 |
| dDBP | -0.43±9.68 | 0(-2.75，5.00) | 0.31 | -1.90±10.74 | -2 (-6,2)^#^ | 0.25 |
| nDBP | -4.11±10.06^##^ | -5.41±10.63^##^ | 0.19 | -4.80±10.52^##^ | -6.98±10.33^##^ | 0.10 |
| 24hMBP | -1.95±10.07 | -3.30±9.32^##^ | 0.35 | -3.02±10.27^#^ | -4.80±9.49^##^ | 0.26 |
| dMBP | -0.42±10.21 | -1 (-7，3) | 0.27 | -1.38±10.43 | -3.28±9.49^#^ | 0.20 |
| nMBP | -4.21±12.77^#^ | -7.38±13.46^##^ | 0.23 | -5.16±13.55^##^ | -8.10±11.93^##^ | 0.17 |

Compared with this group before treatment, #, *P* < 0.05, ##, *P* < 0.01.. 24hSBP: Mean value of 24-hour systolic blood pressure; dSBP: Mean value of daytime systolic blood pressure; nSBP: Mean value of noctural systolic blood pressure; 24hMBP: Mean value of 24-hour diastolic blood pressure; dMBP: Mean value of daytime diastolic blood pressure; nMBP: Mean value of noctural diastolic blood pressure.

**Table S9 Changes in ABPM blood pressure of CCB stratified analysis in PPS**

| **Items** | **CCB** | | | **WITHOUT CCB** | | |
| --- | --- | --- | --- | --- | --- | --- |
|  | **Test (n=33)** | **Control (n=31)** | ***P*** | **Test (n=19)** | **Control (n=20)** | ***P*** |
| 24hSBP | 0.64±9.97 | -6±19.98 | 0.10 | -6.89±8.27^##^ | -3.5±11.31 | 0.29 |
| dSBP | 2.85±9.75 | -4.32±20.11^#^ | 0.07 | -6±8.41^##^ | -2.3±11.13^#^ | 0.25 |
| nSBP | -2.48±12.45 | -9.29±21.38^#^ | 0.12 | -9.37±12.07^##^ | -7.3±15.12 | 0.64 |
| 24hDBP | 0.72±6.46 | -3.6±9.08 | 0.09 | 5.32±4.44^##^ | 4.05±5.44^##^ | 0.25 |
| dDBP | 1.39±8.95 | -0.93±7.54 | 0.08 | -7.63±11.39^##^ | -4.1±7.01^#^ | 0.25 |
| nDBP | -3±10.64 | -8.26±13.28^##^ | 0.09 | -10.05±12.67^##^ | -7±8.85^##^ | 0.40 |
| 24hMBP | 0.06±9.59 | -4.5±10.89^#^ | 0.04* | -8.37±9.36 | -5.25±7.13^##^ | 0.25 |
| dMBP | 2.03±9.5 | -2.6±10.79 | 0.04* | -1.21±1.55^##^ | -0.75±1.37^##^ | 0.27 |
| nMBP | -1.94±8.49 | -9.84±15.49^##^ | 0.04* | -8.05±17.33^##^ | -7.8±10.19^##^ | 0.96 |

Compared with this group before treatment, #, *P* < 0.05, ##, *P* < 0.01. Compared the differences before and after treatment between the two groups, *, *P* < 0.05. CCB, Calcium Channel Blockers. 24hSBP: Mean value of 24-hour systolic blood pressure; dSBP: Mean value of daytime systolic blood pressure; nSBP: Mean value of noctural systolic blood pressure; 24hMBP: Mean value of 24-hour diastolic blood pressure; dMBP: Mean value of daytime diastolic blood pressure; nMBP: Mean value of noctural diastolic blood pressure.

**Table S10 Changes in vital signs (FAS)**

| **Items** | **Test (n=62)** | **Control (n=64)** |
| --- | --- | --- |
| **Breathe** |  |  |
| Baseline | 16.29±2.15 | 15.96±2.46 |
| Treat 4 weeks | 16.94±1.98 | 16.25±1.86 |
| Treat 8 weeks | 16.73±1.88 | 16.25±1.61 |
| Treat 12 weeks | 16.42±1.87 | 16.94±1.82 |
| **Body temperature** |  |  |
| Baseline | 36.31±0.2 | 36.32±0.19 |
| Treat 4 weeks | 36.3±0.22 | 36.27±0.2 |
| Treat 8 weeks | 36.27±0.2 | 36.28±0.23 |
| Treat 12 weeks | 36.23±0.17 | 36.28±0.21 |
| **Heart rate** |  |  |
| Baseline | 71.02±9.94 | 72.63±10.58 |
| Treat 4 weeks | 73.04±8.28 | 74.02±7.93 |
| Treat 8 weeks | 72.56±7.94 | 72.69±7.81 |
| Treat 12 weeks | 72.13±10.88 | 72.61±8.84 |

**Table S11 Changes in laboratory test indexes (FAS)**

| **Items** | **Test (n=62)** | **Control (n=64)** | **Statistic** | ***P*** |
| --- | --- | --- | --- | --- |
| **Alanine transaminase** |  |  |  |  |
| Baseline | 27.52±13.98 | 30.20±17.82 | -0.94 | 0.35 |
| Treat 12 weeks | 23.68±10.36 | 22.51±11.95 |  |  |
| Difference value | -4.12±12.14 | -7.21±18.52 | 0.57 | 0.57 |
| Statistic | 2.56 | 2.99 |  |  |
| *P* | 0.01^##^ | 0.00^##^ |  |  |
| **Aspartate transaminase** |  |  |  |  |
| Baseline | 27.24±11.15 | 28.39±8.86 | -0.64 | 0.52 |
| Treat 12 weeks | 23.65±7.29 | 24.29±8.73 |  |  |
| Difference value | -4.08±10.97 | -4.36±10.19 | -0.43 | 0.67 |
| Statistic | 2.81 | 3.29 |  |  |
| *P* | 0.01 ^##^ | 0.00^##^ |  |  |
| **Creatinine** |  |  |  |  |
| Baseline | 66.58±20.86 | 70.25±49.27 | -0.54 | 0.60 |
| Treat 12 weeks | 66.18±17.59 | 63.41±15.67 |  |  |
| Difference value | -0.31±8.85 | -5.22±49.89 | 0.9 | 0.37 |
| Statistic | 0.27 | 0.8 |  |  |
| *P* | 0.79 | 0.43 |  |  |
| **Urinary microalbumin** |  |  |  |  |
| Baseline | 42.68±74.79 | 52.75±106.53 | -0.61 | 0.54 |
| Treat 12 weeks | 44.5±97.14 | 40.21±71.3 |  |  |
| Difference value | 4.31±55.33 | -12.22±98.44 | 0.27 | 0.79 |
| Statistic | -0.59 | 0.95 |  |  |
| *P* | 0.56 | 0.34 |  |  |
| **Retinol-binding protein** |  |  |  |  |
| Baseline | 51.05±17.7 | 53.9±18.25 | -0.89 | 0.38 |
| Treat 12 weeks | 50.47±15.33 | 50.79±15.18 |  |  |
| Difference value | -0.05±9.59 | -3.36±17.07 | -0.11 | 0.91 |
| Statistic | 0.04 | 1.51 |  |  |
| *P* | 0.97 | 0.14 |  |  |

Compared with this group before treatment, ##, *P* < 0.01.

**Table S12 Adverse event report**

| **Subject ID** | **Group** | **Adverse Event Description** | **Management** | **Outcome** | **Adverse Event Evaluation** | **Related to Investigational Product** |
| --- | --- | --- | --- | --- | --- | --- |
| 1002 | Test | On 10/15/2021, experienced cold and runny nose | Self-purchased (old Relief Tablets | Telephone follow-up on 10/17/2021, symptoms improved and medication was discontinued | AE | No |
| 1004 | Test | On 10/20/2021, experienced occasional chest tightness | Self-purchased Heart-Clearing Pills | Telephone follow-up on 10/22/2021, symptoms relieved | AE | No |
| 1006 | Test | On 10/18/2021, experienced eye bleeding; hospital visit confirmed retinal macular hemorrhage | Oral Thrombolysis Capsules and Iodinated Lecithin | Telephone follow-up on 10/20/2021, symptoms relieved; instructed to continue medication as prescribed, medication discontinued on 11/02/2021 | AE | No |
| 1008 | Test | On 08/17/2021, sudden dizziness and palpitations; self-measured low blood pressure (specific value unknown), considered hypotensive reaction after adding investigational product | Discontinued investigational product | Telephone follow-up on 08/18/2021, dizziness and palpitations disappeared; self-measured BP returned to 140-145/80-85 mmHg; patient requested to withdraw from the clinical trial | AE | Yes |
| 1012 | Control | On 10/06/2021, hyperuricemia diagnosed during physical examination | Oral colchicine | Telephone follow-up on 10/13/2021, no special discomfort; instructed to continue medication as prescribed | AE | No |
| 1014 | Test | On 11/30/2021, experienced cold with sore throat | Self-purchased cold medicine (details unknown) | Telephone follow-up on 12/02/2021, symptoms improved and medication was discontinued | AE | No |
| 1017 | Test | On 12/20/2021, experienced nasal congestion and runny nose after a cold | Self-purchased cold medicine (details unknown) | Telephone follow-up on 12/22/2021, symptoms improved and medication was discontinued | AE | No |
| 2004 | Test | On 11/13/2021, experienced nasal congestion and runny nose after a cold | Self-purchased cold medicine (details unknown) | Telephone follow-up on 11/15/2021, symptoms improved and medication was discontinued | AE | No |
| 3005 | Test | On 11/20/2021, visited doctor for neck pain, diagnosed with cervical spondylosis | Prescribed Jingfukang Capsules | Telephone follow-up on 11/22/2021, symptoms improved; medication continued until the end of the visit period | AE | No |
| 3016 | Test | On 11/25/2021, experienced nasal congestion, runny nose, etc. after a cold | Self-purchased moxifloxacin and compound paracetamol and amantadine capsules | Telephone follow-up on 11/27/2021, symptoms improved; medication discontinued on 11/20/2021 | AE | No |
| 3054 | Test | On 10/22/2021, after taking the drug, self-measured blood pressure showed significant fluctuations | Discontinued investigational product | Telephone follow-up on 10/23/2021, blood pressure values returned to normal; patient requested to withdraw from the clinical trial | AE | Yes |
| 3059 | Test | On 10/20/2021, sudden dizziness; self-measured low blood pressure (specific value unknown), considered hypotensive reaction after adding investigational product | Discontinued investigational product | Telephone follow-up on 10/21/2021, dizziness disappeared; self-measured BP returned to 135-140/80-85 mmHg; patient requested to withdraw from the clinical trial | AE | Yes |
| 3090 | Control | On 10/03/2021, sudden acute myocardial infarction | Emergency hospital treatment | Death on 10/07/2021 | SAE | No |

AE, Adverse Event; SAE, Serious Adverse Event.


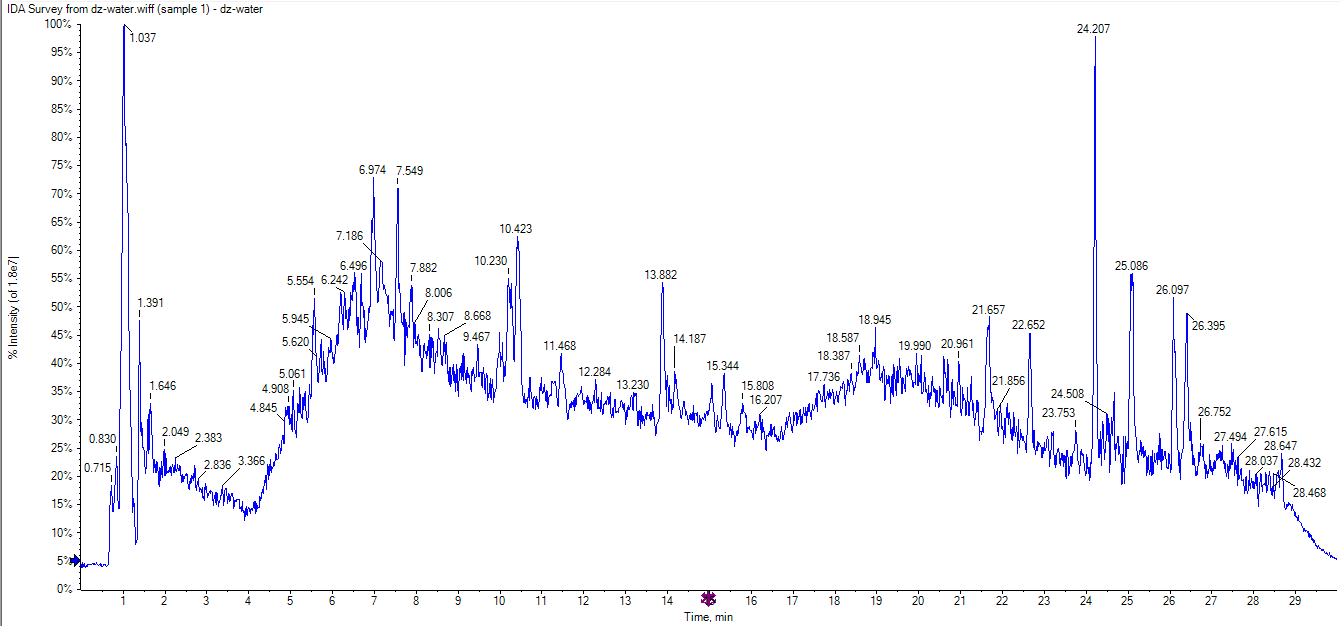


Figure S1 TIC of Quanduzhong capsules.


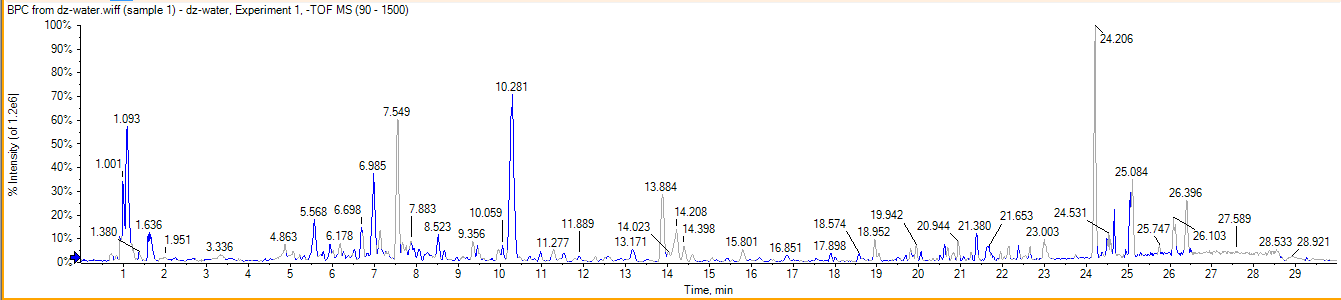


Figure S2 BPC of Quanduzhong capsules.
